# Evaluation of the Prognostic Value of Red Cell Distribution Width to Total Serum Calcium Ratio in Patients with Acute Pancreatitis

**DOI:** 10.1155/2021/6699421

**Published:** 2021-07-27

**Authors:** Tian-Yong Han, Tao Cheng, Bo-Fu Liu, Ya-Rong He, Pan Pan, Wen-Tao Yang, Qiang-Lai, Yu Cao

**Affiliations:** Emergency Department, West China Hospital, Sichuan University, Laboratory of Emergency Medicine, West China Hospital, Sichuan University, Disaster Medical Center, Sichuan University, Chengdu, Sichuan, China

## Abstract

**Introduction:**

Acute pancreatitis (AP) is a sudden inflammatory process in the pancreas with variable involvement of nearby organs or other organ systems, and it is a common cause for hospitalization of gastrointestinal origin. Early prediction of the prognosis of patients with AP is important to help physicians triage the patients and decrease mortality. Red cell distribution width (RDW) and total serum calcium (TSC) have been reported to be useful predictors of the severity of AP, but if these parameters are associated with the prognosis of AP is unknown. The objective of the study was to evaluate whether RDW/TSC can be used to predict the prognosis of patients with AP at an early stage.

**Methods:**

We retrospectively enrolled AP patients admitted to the emergency department of West China Hospital of Sichuan University from January 1, 2016, to June 30, 2016. According to the prognosis, AP patients were divided into ICU group and non-ICU group, surgery group and nonsurgery group, and hospital survival group and hospital death group. Demographic information and clinical and laboratory parameters of all enrolled patients after being admitted to ED were compared between the groups. The receiver operator characteristic (ROC) curves were used to evaluate the prognostic values of RDW, TSC, and RDW/TSC in patients with AP.

**Results:**

A total of 666 AP patients were enrolled in this study, with an average age of 47.99 ± 14.11 years, including 633 patients who survived to discharge and 33 patients who died during hospitalization. The areas under the curve (AUC) of RDW and RDW/TSC predict that patients need to be admitted to ICU (0.773 vs. 0.824 vs. 0.723), patients need surgery treatment (0.744 vs. 0.768 vs. 0.690), and patients survived to hospital discharge (0.809 vs. 0.855 vs. 0.780) were greater than that of TSC, with RDW/TSC being the greatest.

**Conclusions:**

RDW/TSC may be a new method to identify the AP patients who need to be transferred to the ICU, accompanying complications which need surgery treatment, or may be died in hospital at an early stage, and we should pay more attention to RDW/TSC in patients with AP, for they may have a worse prognosis.

## 1. Introduction

Acute pancreatitis (AP) is one of the leading causes of hospitalization among gastrointestinal diseases worldwide [1]. Pancreatitis can be caused by a variety of pathogenesis and may trigger the systemic inflammation response [2]. Based on the revised Atlanta classification, the severity of AP is classified by 3 degrees: mild acute pancreatitis (MAP), moderately severe acute pancreatitis (MSAP), and severe acute pancreatitis (SAP) [3]. According to the severity, the mortality rate of acute pancreatitis varies between 2% and 20% [4–6]. Although patients with MAP may recover spontaneously within a few days, patients with MSAP or SAP may suffer systemic organic complications, which brings a huge challenge to treatment [4, 6–9]. Studies have shown that the first 48 hours after the onset of illness is very important for identifying patients who are at risk of developing systemic complications or death. For patients at risk of death, timely treatment of fluid resuscitation, pain control, and nutritional support may help improve the prognosis of patients [10].

Currently, there are many scoring systems and biomarkers used to predict the prognosis of AP, such as the Ranson criteria [11], Acute Physiology and Chronic Health Evaluation II (APACHE II) [12], Balthazar grade [13, 14], C-reactive protein (CRP), and procalcitonin (PCT). However, the scoring systems have many indicators and the calculations are often complex, which limit their clinical application [11, 12]. Currently, there are no biomarkers that can be used for accurate prognosis prediction. Therefore, the early identification of severity and prognosis of patients with AP remains a great challenge, and it is necessary to predict the prognosis of AP at an early stage.

Red cell distribution width (RDW) is a routine parameter of the complete blood count test, described as simple, easy, inexpensive, and quantitative that measures the size heterogeneity of peripheral red blood cell (RBC) [15–17]. Studies have shown that RDW is not only widely used in blood system diseases, cardiovascular and cerebrovascular diseases, and lung disease but also may be an inflammatory status, such as C-reactive protein, interleukin-6, and fibrinogen, and it closed to AP and may be used to predict prognosis of patients with AP [18–23]. Many studies have shown that low total serum levels of calcium (TSC) play an important role in patients with severe AP and may be used to predict the prognosis of AP patients [24, 25]. Therefore, we designed the study to identify whether RDW/TSC can be used for the early identification of AP patients with poor prognosis.

## 2. Materials and Methods

### 2.1. Participants

Adult patients diagnosed with AP admitted to the emergency department of West China Hospital of Sichuan University from January 1, 2016, to June 30, 2016, were enrolled. AP was diagnosed and stratified according to the 2012 Atlanta classification criteria [3]. The diagnosis of AP required two of three features: (1) prolonged abdominal pain characteristic of AP, (2) threefold elevation of serum amylase and/or lipase levels above the normal range, and (3) characteristic findings of AP on abdominal ultrasonography and/or CT scan. Patients with AP were divided into mild AP (MAP), moderately severe AP (MSAP), and SAP. MAP was defined as an absence of organ failure and an absence of local or systemic complications; MSAP was defined as no evidence of persistent organ failure, but the presence of local or systemic complications and/or organ failure was resolved within 48 hours; SAP was defined as persistent organ failure (>48 hours). Exclusion criteria for the study were as follows: (1) age<18 years; (2) chronic pancreatitis; (3) malignant tumor comorbidity; (4) AP caused by poisoning, surgical operations, and trauma; (5) postoperative pancreatic lesions; (6) women during pregnancy or perinatal period; (7) blood diseases; (8) complicated with chronic diseases and liver and kidney function insufficiency; (9) incomplete clinical data; and (10) missing follow-up.

### 2.2. Data Collection and Ethics Statement

Demographic information and clinical and laboratory parameters of all enrolled patients after admission to the emergency department were collected. Patients with AP were followed up, and the rate of admission to ICU and rate of complications which require surgical treatments and hospital mortality were recorded. The data recording was supervised by the medical team leader, and the data were double-checked to ensure the accuracy. Wrong telephone numbers, three unanswered calls at different times, or not being amenable to follow-up was defined as lost to follow-up. The retrospective cohort study complied with the Declaration of Helsinki and was approved by the Institutional Review Board and Medical Ethics Committee of West China Hospital (Chengdu, China). The requirement for written informed consent was waived because of the retrospective design of this study.

### 2.3. Statistical Analysis

All analyses were performed using the SPSS statistical software version 26.0 (IBM Corporation, Armonk, NY). Continuous variables were presented as the mean ± standard deviation and compared using independent-sample *t*-tests for the normally distributed data. For skewed distributions, the data are presented as the median (interquartile range) and compared using Mann–Whitney *U* nonparametric test. Categorical variables were presented as frequencies (proportions) and compared using the chi-squared or Fisher's exact test as appropriate. The receiver operating characteristic (ROC) curves were used to evaluate the predictive values of RDW, total serum calcium (TSC), and RDW/TSC for the prognosis of patients with AP. The area under the curve (AUC) >0.75 was defined as a good predictive value. Statistical significance was considered when the *P* value < 0.05.

## 3. Results

### 3.1. Clinical Characteristics of the Participants

A total of 666 AP patients were enrolled in this study, with an average age of 47.99 ± 14.11 years, including 431 males (64.7%), and 518 patients with MAP, and 148 patients with MSAP or SAP. Baseline characteristics and clinical features of the two group patients are shown in Table 1.

### 3.2. RDW, TSC and RDW/TSC of AP Patients with Different Prognosis

The RDW, TSC, and RDW/TSC of patients with poorer prognosis who need be admitted to ICU, surgery, or died in hospital were higher than that of patients with better prognosis who need not be admitted to ICU, surgery, or survived to hospital, respectively (Table 2).

### 3.3. ROC Curves Analysis for Predicting Prognosis of AP

**Figure 1:**
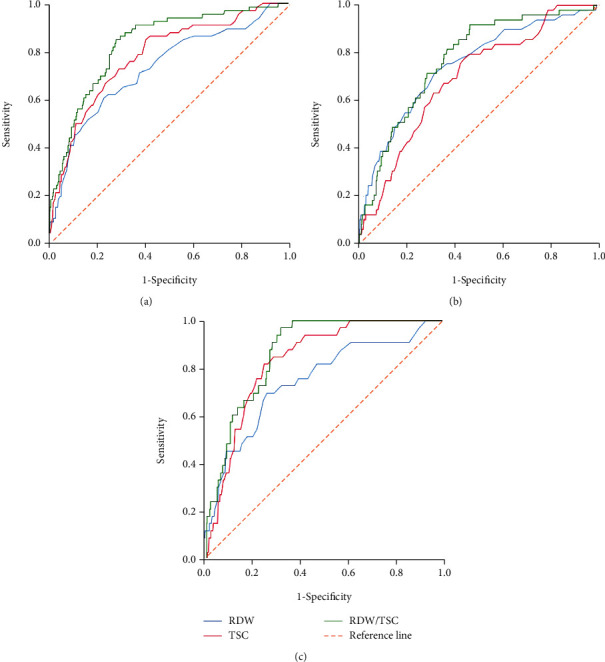
(a) ROC curves analysis for predicting admission to ICU by of RDW, TSC, and RDW/TSC. (b) ROC curves analysis for predicting surgery need by of RDW, TSC, and RDW/TSC. (c) ROC curves analysis for predicting hospital mortality by of RDW, TSC, and RDW/TSC.

These patients were stratified into ICU group (*n* = 66) and non-ICU group (*n* = 600), according to whether the patient was admitted to ICU. The AUCs of RDW, TSC, and RDE/TSC for predicting admission to ICU were 0.723, 0.773, and 0.824, respectively (Figure 1(a) and Table 3). According to whether the patient needs surgery, the patients were stratified into surgery group (*n* = 49) and nonsurgery group (*n* = 617). The AUCs of RDW, TSC, and RDW/TSC for predicting the complications which need surgery treatment were 0.744, 0.690, and 0.768, respectively (Figure 1(b) and Table 3). The patients were stratified into surviving group (*n* = 633) and nonsurviving group (*n* = 33), according to whether the patient survived or died. The AUC of RDW, TSC, and RDE/TSC for predicting hospital mortality were 0.780, 0.809, and 0.855, respectively (Figure 1(c) and Table 3).

## 4. Discussion

As we all know, severe AP cases are usually accompanied by severe complications and high mortality and pay huge medical expenses. In this sense, early detection of the severity of AP patients who may need to be transferred to the intensive care unit (ICU) is critical, not only for improving clinical outcomes but also for decreasing hospitalisation cost [10]. Over years, several scoring systems and biomarkers, such as the Ranson criteria [11], APACHE II [12], Balthazar grade [13, 14], CRP, and PCT, have been well established to predict the severity and hospital mortality of AP. However, the scoring systems involve much examinations that limit their applicability and are cumbersome to operate, leading to most of them be time-consuming and not convenient for clinical practice [11, 12], and the biomarkers are consistently accurate, play a definitive role, or have widespread applicable value, and they cannot predict the complications which need surgery treatment. For example, the Ranson criteria require 48 hours to complete, which will miss the potentially valuable early treatment. Therefore, the early identification of the severity and prognosis of patients with AP remains a great challenge.

Red cell distribution width (RDW) is a routine parameter of the complete blood count test, described as simple, easy, inexpensive, and quantitative that measures the size heterogeneity of peripheral red blood cell (RBC) [15–17]. Many studies have reported that RDW is not only widely used in diseases of the hematological system but also closely related to cardiovascular and cerebrovascular diseases, lung diseases, and infectious disease, which can be used as an indicator to judge the severity of the condition of critically ill patients and a predictor of mortality [26–28]. Previous studies have been reported that it may be used to predict severity and mortality in patients with AP [19–22]; however, the mechanisms underlying these interactions remain ambiguous. There may be several possible mechanisms to explain this. It is well established that the activation of the inflammatory cascade mediated by various inflammatory mediators is the key causes of acute pancreatitis aggravation [29]. Researches have shown that the high RDW is related to elevated levels of several inflammatory markers, such as C-reactive protein (CRP) and interleukin (IL) levels [30]. A possible correlation between the RDW and the severity and characteristics of inflammation may exist, and therefore, it could be used as a parameter that indicates inflammation. Meanwhile, AP can increase inflammatory cytokines that inhibit erythropoietin-induced erythrocyte maturation, which may lead to an elevation in RDW [31].

Difference from other studies on the relationship between RDW and AP, we added serum calcium to evaluate the severity of acute pancreatitis. Studies have shown that hypocalcemia could be a poor prognostic indicator in acute pancreatitis. Currently, the development of hypocalcemia has been included in the prognostic scoring system for AP [15]. Disturbances in calcium homeostasis are fundamental to the pathophysiology of acute pancreatitis [32]. Hypocalcemia in patients with AP often indicates the occurrence of pancreatic necrosis which indicate the possibility of SAP. Although the exact mechanism for the development of hypocalcemia in AP is not clear, most researchers believe that the intra-acinar activation of trypsinogen may lead to the production of free fatty acids by self-digestion, which is related to the saponification of calcium binding [33]. In our study, the main findings were that higher levels of RDW/TSC were observed in patients who were admitted to ICU compared with patients who need not be admitted to ICU, patients who need surgery compared with patients who need not surgery, and nonsurvivors compared with survivors. The AUCs of RDW and RDW/TSC were greater than that of TSC, with RDW/TSC being the greatest, meaning that RDW/TSC has superior predictive value for the prognosis of patients with AP and it may ultimately make it possible to predict the prognosis of patients with AP in the early stage.

However, our study also has several limitations: First, it was a retrospective study in which selection bias potentially existed, and some details regarding clinical or laboratory information might not have been well documented. Second, as a retrospective study, we did not study the changes in these biomarkers at different time points, which may be more predictive for prognosis were deficient. In addition, it was based on a single-center design in our hospital, and it may have some sources of bias and confusion. For a more robust conclusion and to define a precise cut-off value of RDW/TSC, we recommend further multicenter prospective trials with a larger sample size.

## 5. Conclusion

In conclusion, RDW/TSC may be an easily obtained, inexpensive, and useful indicator which can be used to identify the AP patients who need to be transferred to the ICU, accompanying complications which need surgery treatment, or may be died in hospital at an early stage and we should pay more attention to the patient with AP who has an increased RDW/TSC, for they may have a worse prognosis. However, more multicenter prospective studies with a larger sample size are warranted to validate our results.

## Figures and Tables

**Table 1:** Characterization of population according to severity of AP.

Variables	MAP (*n* = 518)	MSAP and SAP (*n* = 148)	Total patients (*n* = 666)	*P* value
Age, year	46.9 ± 13.9	51.9 ± 14.2	47.99 ± 14.11	<0.001^#^
Sex, M/F	343,66.2%	88,59.5%	431, 64.7%	0.129^∗^
Fever, °C	36.58 ± 0.49	36.94 ± 0.67	36.66 ± 0.55	<0.001^#^
HR, beats/min	111.20 ± 419.00	115.59 ± 20.35	112.18 ± 396.57	0.899^#^
RR, beats/min	20.76 ± 2.52	24.92 ± 6.36	21.68 ± 4.10	<0.001^#^
MBP, mmHg	116.52 ± 36.91	112.64 ± 19.90	115.66 ± 33.90	0.22^#^
SPO_2_, %	96.52 ± 2.89	94.87 ± 3.57	96.16 ± 3.13	<0.001^#^
WBC, 10^9^/L	11.14 ± 5.14	13.53 ± 5.64	11.67 ± 5.34	<0.001^#^
PLT,10^9^/L	169.69 ± 80.66	157.85 ± 93.7	167.06 ± 83.82	0.13^#^
Hb, g/L	133.64 ± 22.43	121.18 ± 35.13	130.87 ± 26.28	<0.001^#^
HCT, %	0.48 ± 1.79	0.37 ± 0.10	0.46 ± 1.58	0.452^#^
RDW, %	13.53 ± 1.08	14.78 ± 1.58	13.80 ± 1.31	<0.001^#^
TBIL, *μ*mol/L	25.75 ± 27.72	34.18 ± 42.42	27.63 ± 31.77	0.004^#^
ALB, mmol/L	38.48 ± 5.31	31.20 ± 4.84	36.85 ± 6.03	<0.001^#^
BUN, mmol/L	10.03 ± 5.23	18.59 ± 16.32	11.93 ± 9.63	<0.001^#^
Cr, mmol/L	65.75 ± 25.46	128.76 ± 130.75	79.90 ± 70.82	<0.001^#^
Glu, mmol/L	8.16 ± 3.76	10.48 ± 4.91	8.68 ± 4.16	<0.001^#^
LIP, U/L	299.86 ± 366.26	358.49 ± 401.43	312.76 ± 374.75	0.099^#^
AMY, U/L	264.44 ± 347.86	409.08 ± 479.79	296.21 ± 385.06	<0.001^#^
TSC, mmol/L	2.12 ± 0.18	1.77 ± 2.74	2.04 ± 0.25	<0.001^#^

^∗^ *χ* ^2^ test; ^#^Kruskal-Wallis H-text. HR: heart rate; RR: respiratory rate; MBP: mean blood pressure; WBC: white blood cell; PLT: platelet; Hb: hemoglobin; HCT: hematocrit; RDW: red blood cell distribution width; TBIL: total bilirubin; ALB: albumin; BUN: blood urea nitrogen; Cr: creatinine; Glu: glucose; LIP: lipase; AMY: amylase; TSC: total serum calcium.

**Table 2:** The differences of RDW, TSC, and RDW/TSC according to different prognoses.

Parameter	RDW (%)	TSC (mmol/L)	RDW/TSC
Admission to ICU			
Yes	14.60 (13.60-15.62)	1.85 (1.54-2.02)	8.05 (7.13-9.48)
No	13.50 (12.90-14.20)	2.10 (1.97-2.22)	6.42 (5.96-7.17)
*P*	<0.001	<0.001	<0.001
Need surgery			
Yes	14.10 (13.30-15.33)	2.04 (1.86-2.17)	7.09 (6.40-8.29)
No	13.50 (12.90-14.20)	2.09 (1.95-2.21)	6.46 (5.98-7.29)
*P*	<0.001	0.018	<0.001
Hospital mortality			
Yes	14.6 (13.7-15.8)	1.83 (1.60-1.95)	8.13 (7.20-9.70)
No	13.5 (13.0-14.3)	2.09 (1.96-2.22)	6.46 (5.98-7.29)
*P*	<0.001	<0.001	<0.001

RDW: red blood cell distribution width; TSC: total serum calcium.

**Table 3:** ROC curve analysis of predicting prognosis of patients with AP.

Parameter	AUC	*P* value	95% confidence interval	
			Lower limit	Upper limit
Admission to ICU				
RDW	0.723	<0.001	0.654	0.792
TSC	0.773	<0.001	0.713	0.833
RDW/TSC	0.824	<0.001	0.774	0.875
Need surgery				
RDW	0.744	<0.001	0.669	0.819
TSC	0.690	<0.001	0.618	0.762
RDW/TSC	0.768	<0.001	0.704	0.831
Hospital mortality				
RDW	0.780	<0.001	0.690	0.870
TSC	0.809	<0.001	0.745	0.874
RDW/TSC	0.855	<0.001	0.806	0.904

AUC: area under curve; RDW: red blood cell distribution width; TSC: total serum calcium.

## Data Availability

The data used to support the findings of this study are available from the corresponding author upon request.
